# Performance of artificial intelligence-based software for the automatic detection of lung lesions on chest radiographs of patients with suspected lung cancer

**DOI:** 10.1007/s11604-023-01503-1

**Published:** 2023-11-30

**Authors:** Atsushi Takamatsu, Midori Ueno, Kotaro Yoshida, Takeshi Kobayashi, Satoshi Kobayashi, Toshifumi Gabata

**Affiliations:** 1 Department of Radiology, Kanazawa University Graduate School of Medical Sciences, 13-1 Takaramachi, Kanazawa, Ishikawa 920-8641 Japan; 2 Department of Diagnostic and Interventional Radiology, Ishikawa Prefectural Central Hospital, Kanazawa, Ishikawa 920-8530 Japan

**Keywords:** Artificial intelligence, Deep learning, Automatic detection, Chest radiograph, Lung cancer

## Abstract

**Purpose:**

This study aimed to evaluate the performance of the commercially available artificial intelligence-based software CXR-AID for the automatic detection of pulmonary nodules on the chest radiographs of patients suspected of having lung cancer.

**Materials and methods:**

This retrospective study included 399 patients with clinically suspected lung cancer who underwent CT and chest radiography within 1 month between June 2020 and May 2022. The candidate areas on chest radiographs identified by CXR-AID were categorized into target (properly detected areas) and non-target (improperly detected areas) areas. The non-target areas were further divided into non-target normal areas (false positives for normal structures) and non-target abnormal areas. The visibility score, characteristics and location of the nodules, presence of overlapping structures, and background lung score and presence of pulmonary disease were manually evaluated and compared between the nodules detected or undetected by CXR-AID. The probability indices calculated by CXR-AID were compared between the target and non-target areas.

**Results:**

Among the 450 nodules detected in 399 patients, 331 nodules detected in 313 patients were visible on chest radiographs during manual evaluation. CXR-AID detected 264 of these 331 nodules with a sensitivity of 0.80. The detection sensitivity increased significantly with the visibility score. No significant correlation was observed between the background lung score and sensitivity. The non-target area per image was 0.85, and the probability index of the non-target area was lower than that of the target area. The non-target normal area per image was 0.24. Larger and more solid nodules exhibited higher sensitivities, while nodules with overlapping structures demonstrated lower detection sensitivities.

**Conclusion:**

The nodule detection sensitivity of CXR-AID on chest radiographs was 0.80, and the non-target and non-target normal areas per image were 0.85 and 0.24, respectively. Larger, solid nodules without overlapping structures were detected more readily by CXR-AID.

**Supplementary Information:**

The online version contains supplementary material available at 10.1007/s11604-023-01503-1.

## Introduction

Chest radiography is a basic imaging modality that is used routinely in clinical practice for screening various thoracic diseases owing to its accessibility, cost-effectiveness, and low radiation exposure [1]. Besides its role in screening, chest radiographs find extensive utility in clinics and large hospitals worldwide, catering to a range of clinical scenarios such as diagnosing respiratory infections, monitoring lung disease progression, and assessing trauma-related chest injuries [2, 3]. In general, chest radiographs are interpreted manually by a radiologist or general practitioner. However, the difficulty in maintaining consistent accuracy, as well as the possibility of inaccuracies and missing findings, have become significant sources of concern. Due to inter- and intra-reader variability, the sensitivity of manually detecting pulmonary nodules on chest radiographs varies, ranging from 36 to 84% [4–6].

Various computer-aided detection (CAD) techniques have been proposed for the automatic detection of lesions on chest radiographs [7]. Yet, conventional CAD systems developed before the 2000s showed insufficient performance and were not widely accepted in routine clinical practice. The advances in the field of deep learning technology in recent years have improved the performance of CAD, and the use of artificial intelligence (AI)-based automated diagnosis in clinical practice is increasing [8]. Compared with manual reading, CAD-assisted interpretation improves the detectability of nodules without increasing the false-positive rate on chest radiographs [9, 10]. Several commercially available automatic detection AI software programs approved by the Pharmaceuticals and Medical Devices Agency (PMDA) have been released in Japan; however, few reports have investigated the post-marketing performance of these software programs in the real world [11]. Furthermore, many previous reports have frequently excluded cases with common lung abnormalities—such as pulmonary fibrosis, respiratory tract inflammation, or emphysema—that are often observed on chest radiographs. The exclusion of such abnormalities may result in discrepancies when these software programs are used in daily practice.

The relationship between the detection of pulmonary nodules using AI software and patient background, the characteristics and location of the nodule, and background lung condition is not well known. Therefore, this study aimed to evaluate the performance of the AI-based software CXR-AID for the detection of pulmonary nodules on the chest radiographs of a clinical population and clarify the relationship between the AI software-detected nodules and the patient/nodule characteristics.

## Materials and methods

### Patient selection

The Institutional Review Board of our institution approved this retrospective study and waived the requirement for obtaining informed consent from the patients. The case collection for this study was based on consecutive cases referred to the Department of Respiratory Surgery or Medicine as suspected lung cancer cases with indications for surgery between June 2020 and May 2022 and for which CT scans were performed under the preoperative lung tumor screening protocol at our hospital. The collection process was initiated by searching for diagnostic imaging reports using the name of the relevant protocol as the search term. The CXR data were collected from the nearest dates before and after the CT examination. The cohort of 399 participants in this study had been previously reported in a study that evaluated the performance of other deep learning-based automatic detection software [12].

### Image acquisition

Chest radiographs were acquired using CALNEO HC (DR-ID900, Fujifilm Corporation, Tokyo, Japan), and the imaging parameters were unified (120 kVp, 160 mA, automatic exposure control, grid ratio of 12:1). CT images were acquired using one of the three types of multi-slice CT scanners available at our institution (Light Speed VCT64/Revolution CT, GE Healthcare, Milwaukee, WI, USA; Somatom Definition Flash; Siemens Healthineers, Erlangen, Germany). The scanning and reconstruction parameters of the CT scanners were as follows: voltage, 120 kVp; quality reference, 280 mAs or Noise Index, 9/11; rotation period, 0.4 or 0.5 s; detector collimation, 128 × 0.6 or 64 × 0.6; pitch, 0.508–1.0; and section thickness, 1.25 or 1.5 mm.

### AI software information

The commercially available AI-based software CXR-AID (Fujifilm, Tokyo, Japan), which was approved by the PMDA in 2021, was used in this study. This software automatically detects abnormal lesions as colored overlays and generates a continuous probability index between 0 and 100 corresponding to the probability of nodules, consolidation, and pneumothorax on the chest radiograph. The results are displayed as a color-coded map corresponding to the generated probability index on the chest radiograph. As CXR-AID does not classify abnormal lesions, all detected lesions were included as targets in this study. The maximum probability index of the identified lung-lesion candidates was defined as the probability index of the target.

### Image evaluation

#### Reference standard and performance of AI software

The reference data for the lesions created by a radiologist were developed using the results analyzed in the previous study. The detailed process is as follows [12]. Two radiologists (18 and 9 years of experience) retrospectively reviewed the chest radiographs and corresponding CT images and annotated pulmonary nodules on the chest radiographs with bounding boxes without referring to the results of CXR-AID. The lesion was not annotated if the presence of an abnormal lesion identified on CT could not be confirmed on the chest radiograph. The bounding boxes were annotated after reaching a consensus. We performed annotation and visibility score assessment on the entire lesion for lung cancer showing pneumonia-like findings or lung cancer accompanied by secondary changes in the surrounding areas. In cases with more than four nodules, the top three nodules were selected based on size and visibility scores, as described below. The boundaries of lesion recognition by CXR-AID were identifiable through the extraction of color pixels. In our study, there were no cases in which two nodules were close to each other, as assessed visually by a radiologist. If the center of the final bounding box annotated by the radiologists was within the area segmented by CXR-AID, the area was considered as successfully detecting the nodule, and the lesions identified by CXR-AID were designated as target areas (“true positives” in this study). All other areas identified by CXR-AID were defined as non-target areas (false positives). The probability indices of the target and non-target areas were compared. The Dice similarity coefficient (DSC) and intersection over union (IoU) were calculated to evaluate the extent of interobserver variability in manual segmentation among the radiologists.

#### Nodule evaluation on chest radiographs

Information on nodule characteristics and the background lung condition was obtained from data from a previous study [12]. The following nodule characteristics were evaluated by the two radiologists: nodule type (solid and subsolid), nodule location (craniocaudal and transaxial), and the presence of overlapping/masking structures (clavicle/first rib, hilar vessels, heart, and diaphragm). Nodule visibility (visibility score) was rated on a 4-point scale with reference to the report by Jang et al. [10], with each score indicating the following: 1, very subtle; 2, subtle; 3, moderately visible; and 4, distinctly visible. The background lung status (background lung score) was graded on a 4-point scale with reference to the modified anatomical noise described by De Boo et al. [13], with each score indicating the following: 1, none; 2, mild; 3, moderate; and 4, severe. The visibility and background lung scores for the initial 30 patients were reviewed concurrently by both radiologists, whereas the remaining patients were reviewed independently. In cases of disagreement, the scores were determined by reaching a consensus. One radiologist measured the size of the solid region of the nodule on the CT image. Lung abnormalities like atelectasis, scarring, bronchiolitis, fibrosis, or emphysema were confirmed on the CT image through consensus between the two radiologists. The definitions of the nodule locations, scores, and findings are described in Appendix S1. In cases where surgical intervention was performed, pathology results of the nodules were obtained from the hospital information system.

#### Analysis of the non-target areas

For images with non-target areas, one radiologist (6 years of experience) referred to the CT image to determine the probable cause of detection by CXR-AID. The non-target areas were further classified into two categories: non-target normal areas, where normal structures (pulmonary vessels, bone/cartilage, and hilar structures) were misidentified by CXR-AID; and non-target abnormal areas, where non-neoplastic abnormal findings (scarring, pleural thickening/plaque, fibrosis, and emphysema/bra) were identified by CXR-AID.

### Statistical analysis

Sensitivity analyses were performed on a per-lesion basis. The number of non-target areas and non-target normal areas per chest radiograph was calculated as the total non-target areas or non-target normal areas divided by the number of chest radiographs, respectively. Statistical analyses were performed using R software (version 4.2.1; R Project for Statistical Computing). Nominal variables were compared using the chi-squared test or Fisher’s exact test. Continuous variables, pertaining to patient characteristics and pathological data, were compared using Welch’s two-sample t-test. The Cochran–Armitage trend test was utilized to compare the sensitivity and visibility scores, the sensitivity and background lung scores, as well as the number of non-target areas per image and the background lung score. Univariate logistic regression analysis was performed to identify the factors predictive of detected or undetected nodules and background lung disease. A *p*-value < 0.05 was considered statistically significant for all tests. The agreement between the two readers was calculated using non-weighted kappa statistics. The *Κ-*values were interpreted as follows: poor (*κ* < 0.20), fair (*κ* = 0.21–0.40), moderate (*κ* = 0.41–0.60), good (*κ* = 0.61–0.81), or excellent (*κ* = 0.81–1.00).

## Results

**Fig. 1:**
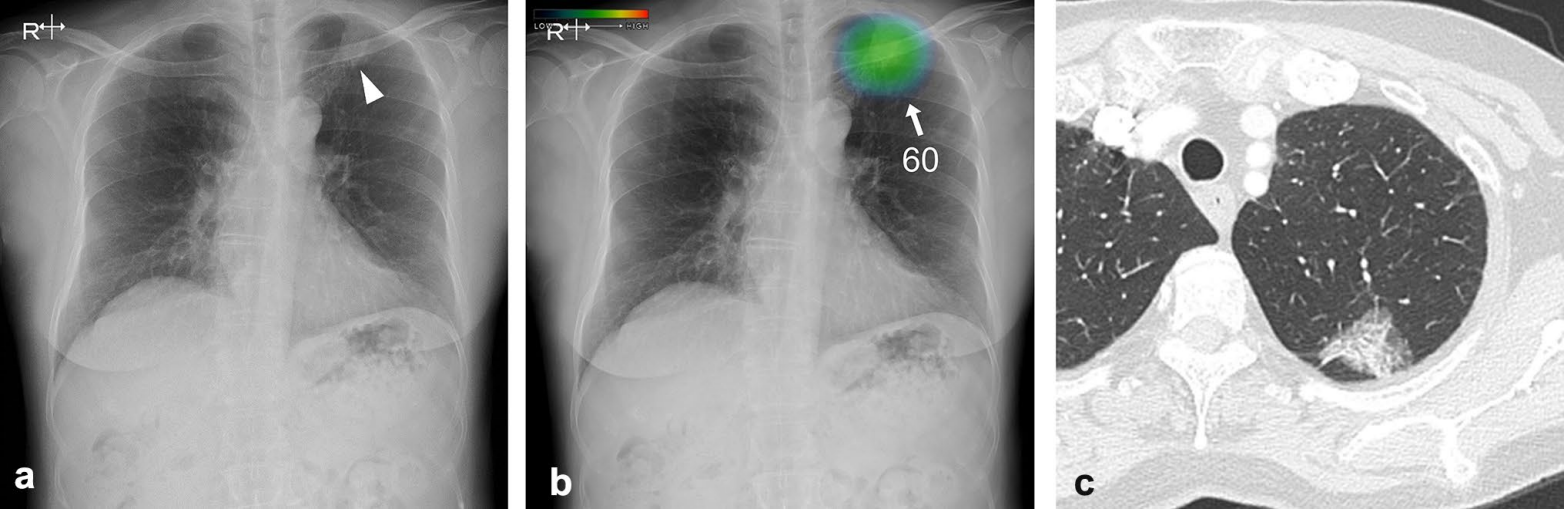
Diagnostic images of 74-year-old female patient with adenocarcinoma in the left upper lung. **a** Chest radiograph showing a nodule in the left upper lung masked by the left clavicle and first rib (arrowhead), with a visibility score of 3 and a background lung score of 2. **b** The nodule was properly detected by CAD with a probability of 60. **c** Chest computed tomography showing a part-solid nodule with a solid part of 2.2 cm in size

**Fig. 2:**
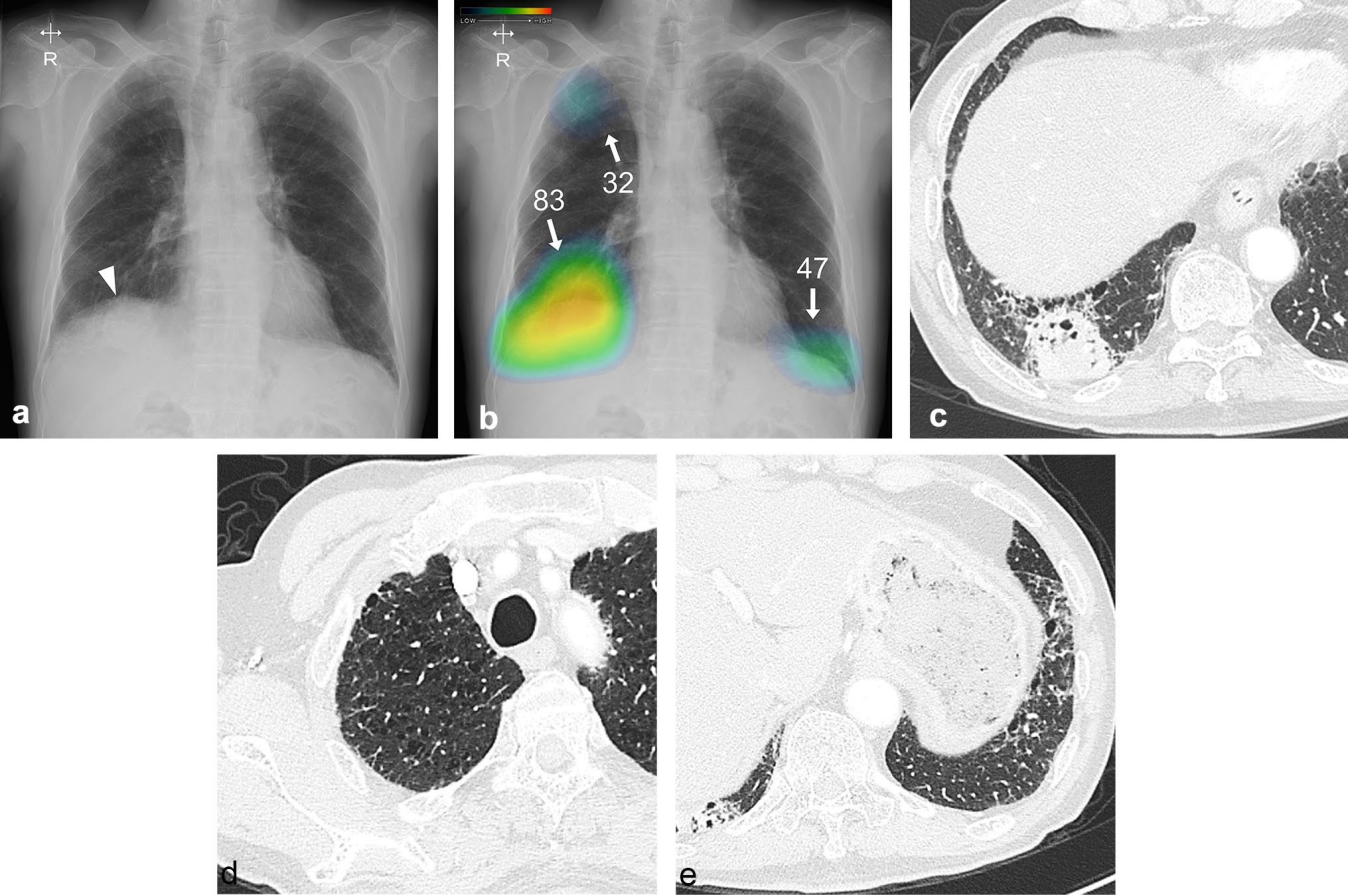
Diagnostic images of a 74-year-old male patient with squamous cell carcinoma in the right lower lung. **a** Chest radiograph showing a nodule in the right lower lung masked by the diaphragm (arrowhead), with a visibility score of 2 and a background lung score of 4. **b** The nodule was properly detected by the CAD with a probability of 83, with two non-target areas were detected with probabilities of 47 and 32, respectively. **c** Chest computed tomography (CT) showing a solid mass measuring 4.6 cm in size. **d** CT did not reveal any abnormality on the lesion with a score of 32, which was considered a non-target normal area. **e** CT revealed fibrosis on the lesion with a score of 47, which was considered a non-target abnormal area

A total of 450 nodules were identified in the CT images of 399 patients (259 men and 140 women; mean age, 71 years; range 26–90 years) and were included in the analysis. Among these 450 nodules, 119 nodules detected in 106 patients were deemed invisible on chest radiographs during manual evaluation and excluded from further analyses. The remaining 331 nodules detected in 314 patients (209 men and 105 women; mean age, 72 years; range 35–90 years) were visible on chest radiographs. The sensitivity of CXR-AID for the detection of nodules was 0.80 (detected nodules, 264; undetected nodules, 67). Table 1 presents the detailed patient characteristics and pathological data. No differences in sex, age, or smoking history were observed between the patients with nodules detected or undetected by CXR-AID; however, the proportion of adenocarcinoma was lesser among the detected lesions. The detection performance of AI software based on subgrouping by nodule size is presented in Supplemental Table 1. Larger nodules exhibited higher detectability by the AI software compared to smaller nodules. Table 2 presents the relationship between nodule detection by AI and the visibility score or background lung score. All nodules with a visibility score of 4 were detected by CXR-AID (62/62 nodules), whereas only 37% (27/73) of the nodules with a visibility score of 1 (very subtle) were detected by CXR-AID. Thus, a higher visibility score was associated with a higher nodule detection sensitivity. A borderline significant difference was observed between nodule detection and the background lung score. In contrast, the number of non-target areas per image increased significantly in patients with higher background lung scores. Representative cases are shown in Figs. 1 and 2.

**Table 1:** Patient characteristics and pathological data of the detected and undetected nodules

	Overall	Detected	Undetected	p-value
	(*n* = 314)	(*n* = 254)	(*n* = 60)	
Sex				> 0.9
Male	209 (67%)	169 (67%)	40 (67%)	
Female	105 (33%)	85 (33%)	20 (33%)	
Age, years, mean (SD)	72 (9)	72 (9)	71 (8)	0.7
Smoking history				0.4
Current smoker	34 (11%)	29 (11%)	5 (8%)	
Former smoker	187 (60%)	154 (61%)	33 (55%)	
Never smoker	92 (29%)	71 (28%)	22 (37%)	
	(*n* = 331)	(*n* = 264)	(*n* = 67)	
Pathology of nodule				0.003^a^
Adenocarcinoma	184 (56%)	136 (52%)	48 (72%)	
Squamous cell carcinoma	63 (19%)	58 (22%)	5 (7%)	
Small cell carcinoma	9 (3%)	9 (3%)	0 (0%)	
The other carcinoma	24 (7%)	21 (8%)	3 (5%)	
No evidence of malignancy	30 (9%)	25 (10%)	5 (7%)	
No pathologic information	21 (6%)	15 (5%)	6 (9%)	

^#a^*P*-value was calculated between the nodules of adenocarcinoma and other carcinomas (squamous cell carcinoma, small cell carcinoma, and other carcinomas)

**Table 2:** Relation of sensitivity of CXR-AID and visibility score or background lung score

	Sensitivity	*p*-value	Non-target area per image	*p*-value
Visibility score		< 0.001		
1	0.37 (27/73)			
2	0.83 (84/101)			
3	0.96 (91/95)			
4	1.00 (62/62)			
Background lung score		0.051		< 0.001
1	0.78 (40/51)		0.28 (23/82)	
2	0.76 (141/186)		0.67 (143/214)	
3	0.88 (51/58)		1.48 (96/65)	
4	0.89 (32/36)		2.02 (77/38)

**Fig. 3:**
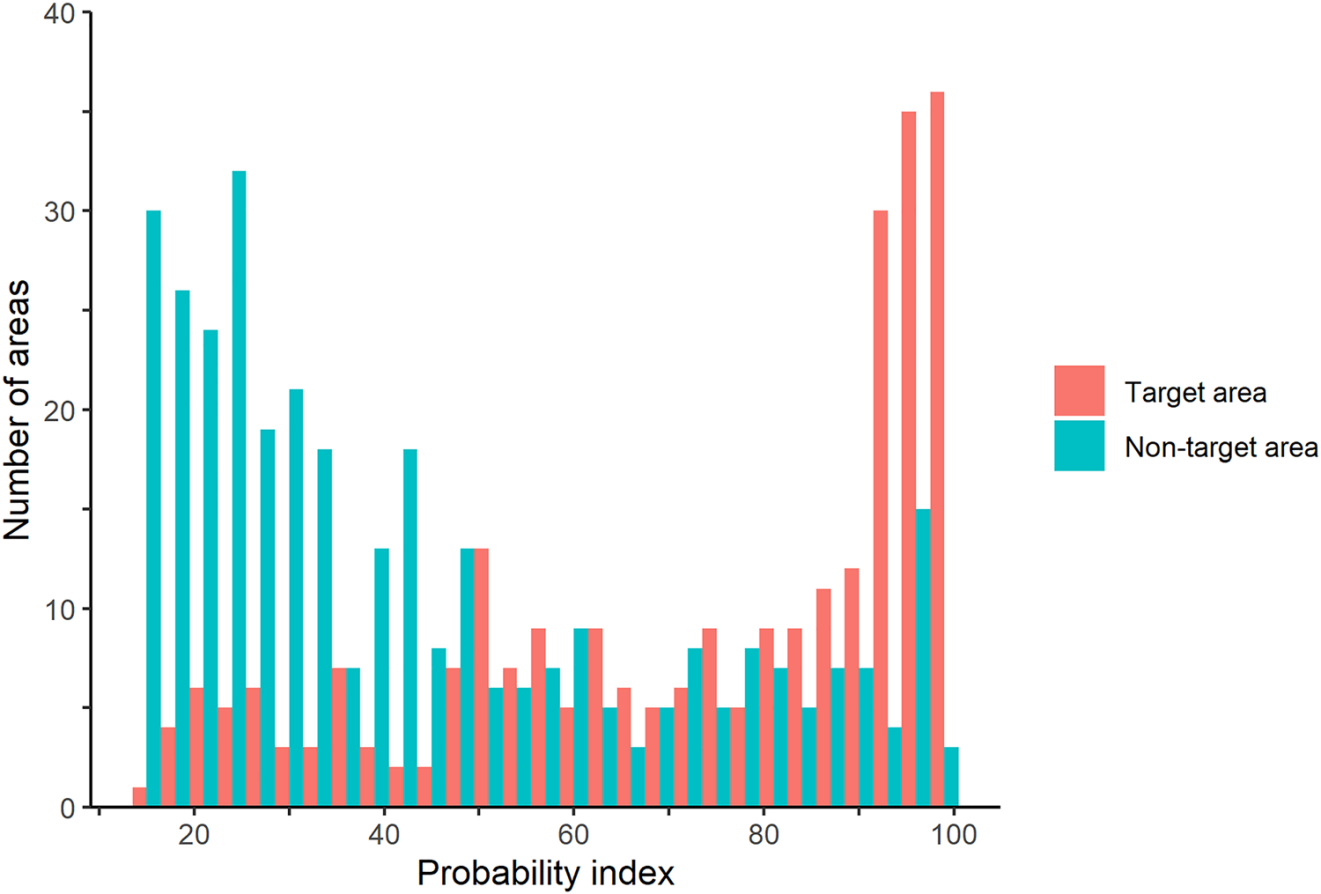
Histogram of the probability index of the target and non-target areas. The X-axis represents the probability index for target and non-target areas. The y-axis represents the number of areas. The probability index of the target areas is significantly higher than that of the non-target areas (*p* < 0.001)

Figure 3 presents the distribution of the probability indices for the target and non-target areas. The mean probability indices of the target and non-target areas were 73.4 (95% confidence interval [CI] 70.4–76.4) and 43.6 (95% CI 40.9–46.3), respectively, indicating a significant difference (*p* < 0.001). Table 3 presents the details of the non-target areas. A total of 339 non-target areas (0.85 per image) were detected in 206 patients. Among these 339 non-target areas, 244 were non-target abnormal areas (0.61 per image), and 95 were non-target normal areas (0.24 per image).

**Table 3:** Details and sub-classification of the non-target areas

	*n* = 339
Non-target abnormal areas	244
Scaring	87
Fibrosis	54
Emphysema/bulla	33
Pleural thickening/plaque	19
Others	51
Non-target normal areas	95
Pulmonary vessels	40
Bone/cartilage	34
Hilar structures	15
Others	6

Table 4 presents the nodule characteristics and presence of background lung disease. The detected nodules were found to be larger in size. Solid nodules were detected more frequently, whereas subsolid nodules were detected less frequently. There was no evidence of differences in the craniocaudal location; however, the nodules detected using CXR-AID were more prevalent on the lateral side. The undetected nodules were located more frequently in areas with overlapping structures, particularly in the hilar vessels.

**Table 4:** Nodule characteristics and presence of background lung disease

	Detected	Undetected	OR (95% CI) ^a^	p-value
	(*n* = 264)	(*n* = 67)		
Solid part size cm, mean (SD)	31 (21)	16 (11)	0.91 (0.88, 0.94)	< 0.001
Nodule type				
Solid nodule	218 (83%)	38 (57%)	Ref	
Subsolid nodule	46 (17%)	29 (43%)	3.62 (2.02, 6.46)	< 0.001
Craniocaudal location				
Right upper lung	67 (25%)	14 (21%)	Ref	
Right middle lung	61 (23%)	15 (23%)	1.18 (0.52, 2.66)	0.7
Right lower lung	39 (15%)	8 (12%)	0.98 (0.36, 2.51)	> 0.9
Left upper lung	45 (17%)	11 (17%)	1.28 (0.53, 3.02)	0.6
Left middle lung	35 (13%)	12 (18%)	1.64 (0.68, 3.94)	0.3
Left lower lung	17 (7%)	6 (10%)	1.69 (0.53, 4.92)	0.3
Transaxial location				
Medial	104 (39%)	39 (58%)	Ref	
Lateral	160 (61%)	28 (42%)	0.47 (0.27, 0.80)	0.006
Presence of pulmonary disease				
Atelectasis	25 (10%)	4 (6%)	0.61 (0.17, 1.63)	0.4
Scaring	61 (23%)	17 (25%)	1.13 (0.60, 2.07)	0.7
Bronchitis	34 (13%)	6 (9%)	0.67 (0.24, 1.55)	0.4
Fibrosis	41 (16%)	6 (9%)	0.53 (0.20, 1.23)	0.2
Emphysema	127 (48%)	24 (36%)	0.60 (0.34, 1.04)	0.073
	(*n* = 267)	(*n* = 69)		
Overlapped/masked structure				
No overlapping/masking structure	161 (60%)	31 (45%)	Ref	
Presence of overlapping/masking structure	106 (40%)	38 (55%)	1.86 (1.09, 3.19)	0.023
Clavicle/first rib	43 (16%)	11 (16%)	1.33 (0.60, 2.79)	0.5
Hilar vessels	40 (15%)	17 (25%)	2.21 (1.10, 4.35)	0.024
Heart	10 (4%)	5 (7%)	2.60 (0.77, 7.86)	0.1
Diaphragm	13 (5%)	5 (7%)	2.00 (0.61, 5.73)	0.2

*OR* odds ratio, *CI* confidence interval, *SD* standard deviation

^a^Represents the odds ratio of each variable for undetected nodules

The agreement between the visibility scores of the two radiologists was strong, with a *κ*-value of 0.68. The agreement between the background lung scores of the two thoracic radiologists was moderate, indicated by a *κ*-value of 0.52.

## Discussion

We evaluated the performance of the commercially available AI software CXR-AID for the detection of pulmonary nodules in clinical cases and the relationship between the detection of pulmonary nodules, their characteristics, and background lungs. The sensitivity of CXR-AID for the detection of visible nodules was 0.80, the non-target area per image was 0.85, and the non-target normal area per image was 0.24. The probability indices of the target areas were higher than those of the non-target areas. Detection sensitivity was observed to be higher for larger and more solid nodules, whereas nodules characterized by overlapping structures exhibited lower rates of detection.

In a previous study by Nam et al. a sensitivity of 0.79–0.91 for nodule detection by AI was reported [14]. Other reports have shown that the sensitivity for the manual detection of pulmonary nodules on chest radiographs varies from 36 to 84% [4–6]. The sensitivity of the present study was equivalent to that reported by a previous study, and the result that most nodules with visibility scores of 3 or 4 (moderately or distinctly visible) were detected by CXR-AID was comparable with manual detection. Compared with the high sensitivity for larger and solid nodules, the sensitivity for smaller nodules, subsolid nodules, or nodules with overlapping structures was low. The detection of these nodules by CXR-AID was found to be inadequate. Therefore, additional management measures are advisable to prevent these nodules from being overlooked. However, it is important to acknowledge that these nodules are challenging to detect even through manual examination.

De Boo et al. reported that anatomic noise related to smoking and age did not lead to a decrease in the nodule detection sensitivity of CAD; however, it was observed to lower the manual detection sensitivity [13]. The present study showed no evidence of a significant relationship; however, a borderline relationship was observed between the background lung condition and the detection of the nodule by CXR-AID. While severe background lung conditions are typically anticipated to diminish manual detection performance their impact on automatic detection by AI is comparatively less [13]. Therefore, AI software could assist in increasing sensitivity in patients with severe background lung disease, in whom radiologists are prone to show decreased performance.

Conventional image-processing-based CAD may result in increased false-positive rates; however, the development of deep learning can solve this problem [13, 14]. The rate of non-target areas was 0.85 per image, indicating a higher value in comparison to that reported in previous studies, where the false-positive rate ranged from 0.1 to 0.3 per image [9, 14]. This may be attributed to CXR-AID targeting consolidations, pneumothorax, and nodules concurrently. Therefore, it might be inappropriate to compare the number of non-target areas per image in this study with the false-positive rates of previous studies. After categorizing the non-target areas into instances of misidentification as abnormal (non-target abnormal areas) or normal findings (non-target normal areas), it is important to note that the number of non-target normal areas per image, which specifically represents the areas identified as false positives, was found to be 0.24. This value accurately reflects the instances of misidentification that result in non-target normal areas, and its compatibility with the study's findings is established. The probability indices of the non-target areas were comparatively lower than those of the target areas. This difference could be valuable for discerning whether the detected areas are indeed true positives or false positives.

This study included consecutive candidates with lung cancer from a single center with no exclusion criteria. Although several reports have investigated the performance of CAD on chest radiographs, most of these studies were based on experimental data that often-included intentional selection bias, such as limiting the size of nodules or excluding cases with severe background lung conditions. Chest radiographs in real-world settings contain a variety of nodules, background lung conditions, and visibility. Therefore, the performance of the previous study may differ from that in the real world. Our dataset, consisting of consecutive clinical cases, is more similar to real-world data than to the experimental data used in previous studies, and the CAD performance in our results is considered to be closer to the actual performance in clinical practice.

The present study was analyzed using cases nearly identical to those in the previous study employing different AI software [12]. The CXR-AID in the present study provides all lesion candidates, including probability information for lesions, whereas the other AI software does not provide the probability information. Therefore, making an accurate comparison with existing AI software that relies on threshold settings is challenging.

This study has certain limitations. First, as this was a single-center study and the image quality of the chest radiographs was relatively consistent, the performance of CXR-AID in other institutions and imaging conditions was not investigated. Second, the participants of this study were patients suspected of having lung cancer and a higher rate of lung lesions than the general patient population. Therefore, the performance in other patient populations, including healthy individuals undergoing medical health checkups, was not evaluated. Third, the pulmonary nodules analyzed in this study included nodules other than those pathologically diagnosed as lung cancer. Although the performance in this study was not strictly based on lung cancer, it was based on consecutive cases of clinically suspected lung cancer using CT as the reference standard, and its performance is relevant to real-world clinical practice. Fourth, assessing the exact number of false positives is impossible because discrimination between non-target normal areas and non-target abnormal areas relies on a manual assessment by a radiologist and cannot be performed objectively. Fifth, this study evaluates the performance of the sole AI software and has not been assessed as a radiologist's assistance. Future evaluation as an interpretation assistant would be desirable. Lastly, the software may be updated in the future, which may result in changes in its performance.

In conclusion, the nodule detection sensitivity of the commercially available AI software CXR-AID was 0.80, the non-target area per image was 0.85, and the non-target abnormal area was 0.24. Larger, solid nodules without overlapping structures were detected more frequently. The findings of this study can establish a valuable benchmark for the practical application of AI software in real-world scenarios.

### Supplementary Information

Below is the link to the electronic supplementary material.

Supplementary file1 (DOCX 24 KB)
