# Supplementary material for: Performance of artificial intelligence-based software for the automatic detection of lung lesions on chest radiographs of patients with suspected lung cancer
Source: Jpn J Radiol. 2023 Nov 30;42(3):291–9. doi: 10.1007/s11604-023-01503-1 (PMC10899395; doi:10.1007/s11604-023-01503-1)
Supplement: Supplementary file 1 — Supplementary file1 (DOCX 24 KB) [file 11604_2023_1503_MOESM1_ESM.docx]

**Appendix S1 Definition of nodule locations, visibility score, background lung condition, and background abnormalities on CT**

The craniocaudal location and transaxial location were based on Nam et al.’s study [2].

The visibility scores were based on the report by Jang et al. and the background lung scores were graded using modified anatomical noise described by De Boo et al. [10, 13].

The background lung abnormalities on CT were evaluated based on the glossary of terms for thoracic imaging by the Fleischner Society and the previous reports [S1-S4].

･ The craniocaudal location

- Upper lung: superior to the carina.
- Middle lung: between the carina and lower lung.
- Lower lung: inferior to the inferior pulmonary vein lung.

･ Transaxial location

- Medial: medial half of the lung.
- Lateral: lateral half of the lung.

･ Visibility score

- 1 (very subtle): visibility intermediate between invisible and score 2.
- 2 (subtle): nodules that could be detected by careful observation.
- 3 (moderately subtle): visibility intermediate between score 2 and 4.
- 4 (well visible): the presence of obvious nodules.

･ Background lung score

- 1 (none): obviously normal lung.
- 2 (mild): limited normal but relatively dirty lung.
- 3 (moderate): abnormal findings in less than 50% of both lungs.
- 4 (severe): abnormal findings diffuse in more than 50% of both lungs.

･ Background lung abnormalities on CT

- Atelectasis: a linear, discoid, platelike lesions and volume reduction accompanied by increased attenuation.
- Scarring: a linear band-like lesion with or without calcification.
- Bronchiolitis: tree-in-bud pattern, centrilobular nodules, and bronchiolar wall thickening.
- Fibrosis: the presence of architectural distortion with traction bronchiectasis or honeycombing.
- Emphysema: focal areas or regions of low attenuation, usually without visible walls.

**Supplemental references**

S1. Liang M, Tang W, Xu DM, et al. Low-dose CT screening for lung cancer: Computer-aided detection of missed lung cancers. Radiology 2016; 281:279-288

S2. Geckel C, Hansell DM. Imaging the 'dirty lung'--has high resolution computed tomography cleared the smoke? Clin Radiol 1998; 53:717–722

S3. Hansell DM, Bankier AA, MacMahon H, et al. Fleischner society: Glossary of terms for thoracic imaging. Radiology 2008; 246:697–722

S4. Hatabu H, Hunninghake GM, Richeldi L, et al. Interstitial lung abnormalities detected incidentally on ct: A position paper from the fleischner society. Lancet Respir Med 2020; 8:726–737

**Supplemental Table 1.** **Relation of the sensitivity of CXR-AID and the size of nodules**

| Size | All | Detected | Undetected | Sensitivity |
| --- | --- | --- | --- | --- |
| < 15 mm | 80 | 42 | 38 | 0.53 |
| 15 - 29 mm | 142 | 116 | 26 | 0.82 |
| ≥ 30 mm | 109 | 106 | 3 | 0.97 |
